# Integrated Digit-in-Noise Test: A Rapid Screening Tool for Hearing and Cognitive Function

**DOI:** 10.1093/geroni/igaf122.4334

**Published:** 2025-12-31

**Authors:** Lena Wong, June Tung, Shangqiguo Wang

**Affiliations:** The University of Hong Kong, Hong Kong, China (People’s Republic); The University of Hong Kong, Hong Kong, China (People’s Republic); The University of Hong Kong, Hong Kong, China (People’s Republic)

## Abstract

A rapid and easy-to-administer screening tool is essential for community-based detection of hearing loss and cognitive decline. The Integrated Digit-in-Noise Test (iDIN) extends the traditional Digit-in-Noise Test (DIN) by incorporating 2- to 5-digit sequences, as the only test to simultaneous assess and differentiate hearing and cognition. Speech Reception Thresholds (SRTs) are measured as the signal-to-noise ratio (SNR) at which 50% of digits are correctly identified. Specifically, 3-digit SRTs are used for hearing screening, while the difference between backward and forward 3-digit SRTs (SRT3b-3) serves as an indicator of cognitive function. In this study, 601 community-dwelling participants with potential but undiagnosed hearing loss were recruited (mean age 76.0 ± 8.8 years; education 6.4 ± 4.4 years; MoCA 21.5 ± 6.4). In terms of hearing screening, the average 3-digit SRT was -4.3 ± 7.0 dB SNR, with a cutoff of -7.7 dB SNR for hearing loss detection (35 dB HL in better ear) (sensitivity 0.85, specificity 0.73). For cognitive screening, the mean SRT3b-3 was 5.03 ± 7.32 dB SNR. Using MoCA thresholds of 21/22 for MCI, the optimal SRT3b-3 cutoff was 3.3 dB (sensitivity 0.74, specificity 0.76); for dementia (MoCA 15), the cutoff was 5.5 dB (sensitivity 0.83, specificity 0.77). No significant correlation was found between SRT3b-3 and better ear hearing levels, indicating that iDIN can effectively be used for cognitive screening in older adults with hearing impairment. In conclusion, iDIN shows promise as a quick, dual-purpose screening tool for early detection of hearing and cognitive issues in community settings.

